# Inclusive EFL Teaching for Young Students with Special Needs: A Case in China

**DOI:** 10.3390/children9050749

**Published:** 2022-05-19

**Authors:** Jinjin Lu, Han Jiang, Yi Huang

**Affiliations:** 1 Department of Educational Studies, Xi’an Jiaotong-Liverpool University, Suzhou 215123, China; jinjin.lu@xjtlu.edu.cn; 2 School of Special Education, Zhejiang Normal University, Hangzhou 311231, China; 3 Department of Psychology, Faculty of Social Studies, Masaryk University, 602 00 Brno, Czech Republic; yihuang@mail.muni.cz; 4 Institute for Research of Children, Youth and Family, Faculty of Social Studies, Masaryk University, 602 00 Brno, Czech Republic

**Keywords:** special education needs, learning in regular classrooms (LRC), EFL teaching and learning, inclusive education training, inclusion regulations

## Abstract

In China, English as a foreign language is important and compulsory from primary education to higher education, essentially because English has become a global language. The Ministry of Education emphasizes that school principals should attempt to train teachers in special education and in assisting students with special education needs (SEN) in regular classes via supportive services. However, EFL teachers usually have insufficient training and do not know how to adjust their teaching methods for students with SEN in regular classes. This study investigated 328 teachers’ teaching practices and their attitudes toward including students with SEN in K–12 English classes in the three largest provinces in east, south, and central China. The findings indicated that English teachers have not used specific teaching resources to teach students with SEN. Teachers noted that they were not provided with specialized training and there were not enough teaching assistants to help the students with SEN. There were significant statistical differences found between primary school teachers and middle school teachers with and without special education training regarding inclusion practices and their attitudes toward inclusion (regarding students with SEN). Most English teachers believe that students with SEN should be taught in special classes with specialized materials rather than in regular EFL classes.

## 1. Introduction

Inclusion is considered a critical issue in discussions on human rights and education equality; it has led to inclusive instructional practices worldwide [1,2,3,4,5,6,7,8]. In China, significant progress has been made in recent decades regarding the development of inclusive education. The policy related to inclusive education has been continuously revised to assist more students with disabilities [9]. The Ministry of Education, in a recent educational reform (i.e., since the 1980s) initiated the ‘Learning in Regular Classrooms’ (LRC) initiative. Moreover, *suibanjiudu* (随班就读) was proposed and implemented in some small regions and less developed cities [10,11]. Xu and colleagues [11] believe that the types of SEN (regarding students) should include intellectual disabilities, visual impairment, and hearing impairment. However, besides the three types of SEN, the LRC policy proposed that physical disabilities, speech and language disabilities, mental disorders (ASD is under this category), and multiple disabilities be included in mainstream classrooms [12]. This policy was documented as an important milestone in the Special Education Promotion Plan [13].

Although the quality and equality of education have improved, the LRC initiative is still lagging, particularly regarding EFL teaching practices. English is an important foreign language in China; it has been a compulsory subject since the restoration of the college entrance examination in China. As EFL education gradually develops in China, many research studies are focusing on Chinese pupils learning English from various aspects, such as EFL learning motivations, e.g., [14,15,16,17], EFL learning styles, e.g., [18,19], EFL teaching strategies, e.g., [20,21,22], language proficiency, e.g., [23,24,25,26], and language policies, e.g., [27,28] in China. However, most of the previous studies focused on EFL students in Chinese universities and teachers’ instructional practices, which involved higher education or students without SEN. Few research studies have focused on investigating instructional practices and English teachers’ attitudes toward the acceptance of young Chinese students with SEN (all categories included) into the LRC initiative. In this case, we examined EFL teachers’ teaching practices, attitudes toward the LRC initiative, and their professional training in terms of supporting students with SEN in a Chinese context. 

### 1.1. The Policy and Practice of LRC

An increasing number of Chinese students with SEN are enrolling in public schools [29]. The number of students with SEN enrolled in special schools has increased by 10.85% since 2020. In China, more than 50% of students with SEN are placed into LRC initiatives in public schools. The LRC initiative is a major form for students with SEN to join public schools. The other forms are home-based teaching and special education (rather than inclusive teaching). According to the National Bureau of Statistics of China [30], the number of students with SEN in LRC initiatives at primary schools was twice the amount compared to middle schools. That is, public primary schools have inclusive teaching; learning MOE [13] requires that students with SEN be encouraged to receive education in inclusive teaching and learning environments, as part of the 14th five-year strategic plan. 

Scholars have not reached a consensus on the definition of inclusive education. Some researchers believe that inclusion refers to the placement of all students in regular schools and classrooms, regardless of their level of ability [31]. Others argue that the definition of SEN is broad and that we need to consider disability categories to include all children who need additional support. Florian [32] noted that it is important to consider the different needs of students with SEN and to further explore how to educate ‘all’. In the Chinese context, the LRC initiative was proposed to be within the umbrella of the global inclusive education program [33]. Despite Piao [34] claiming that this is just a representation of a commonality in the evolution of special education, the commonality of today’s global trend in special education is inclusive education. Most nations, including China, have embraced the idea of inclusive schools and society created by the Salamanca statement, which was released in 1994 and provides these countries with reasons and incentives for establishing objectives and strategies for inclusive education [35]. China’s LRC initiative has been incorporated into the worldwide wave of inclusive education; this shared experience is a result of the internationalization of the LRC initiative. This is also reflected in the Chinese Special Education Improvement Act (SEIA) [13], which puts a lot of effort into assisting more children with SEN, so they could have equal rights to enjoy education in China. 

The characteristics of LRC do not conflict with inclusive education. Inclusive education is still being explored and developed globally. That is, countries (including many developed countries) do not have global success stories regarding the implementation of inclusive education [36]. In other words, each nation needs to develop a model that adapts to its educational system and culture. Due to historical and cultural differences, the promotion process of LRC is not that smooth and still needs a lot of work in rural and remote areas. Xiao [37] described specific methods for implementing and promoting LRC in China. From the document regulations, these essential methods include the following: (1) students with SEN should be provided with the same subjects as other students in inclusive classrooms; they should be assisted by instructors who could help them deal with problems that ordinary classroom teachers are unable to resolve; (2) the enrolment of students with SEN should be in the same fashion as normal students; (3) the teaching materials for students with SEN should be the same as those for ordinary children, but they should be adjusted to students’ unique educational needs; (4) teachers’ qualifications and professional development should be a very rigorous process so they could understand and master specific skills/knowledge about the LRC initiative [37]. Though the plan starts with good intentions, there is a big gap between government policies and practice in schools. There is only one empirical study that was undertaken; Deng [33] found a number of conflicts or gaps between urban and rural areas in central China in terms of the policy and implementation, as well as inadequate resource provision in implementing the LRC programs. Various conflicts were found in previous studies, including gaps between policies and insufficient resource provisions, different understandings from teachers and school principals, an elite selection of examination drilling, quality outcome-based education from a children-centered perspective, and Western and Chinese cultural differences toward inclusion [7,9]. These issues raised Chinese scholars’ concerns about the quality and efficacy of the LRC initiative; researchers tended to view ‘Learning in Regular Classrooms’ as ‘sitting in regular classrooms’ [38,39]. 

### 1.2. Teachers’ Attitudes toward Inclusion 

Teachers’ attitudes toward inclusion have been discussed widely in the West and in China. Researchers believe that teachers’ attitudes and their sustained professional training in terms of specialized knowledge of SEN are important factors. Previous studies found that teachers’ attitudes impacted the learning experiences among pupils with SEN [40,41]. Taylor and Ringlaben [42] argued that teachers’ negative attitudes toward inclusive education would have a significant impact on school culture and, as a result, on student learning performances. Hamre and Pianta [43] evidenced that teachers who have positive attitudes toward their students tend to give them more positive attention, enhancing the students’ feelings of efficacy; this shows the effort invested in providing positive learning experiences. However, practically, teachers tended to have more negative attitudes toward their students with SEN [44], which resulted in negative learning outcomes. Xiao [37] argued that “teachers generally lack basic knowledge and skills in special education in China” [37]. Currently, pre-service teachers who work in regular schools do not need to take lessons or finish professional development programs in special education [12]. This has resulted in a great majority of teachers in regular schools who do not have knowledge about theories and teaching strategies in the special education field. Consequently, this will lead to a number of issues in their daily work lives if they have difficulties in dealing with students with SEN.

### 1.3. EFL Teaching Practices in Inclusive Contexts

EFL is one of the most important subjects in both primary and middle schools in China and in other countries as it connects closely with educational and economic development in the 21st century [45,46]. In Europe, the Teaching Languages at School in Europe provides a comprehensive overview of language learning, teacher training and qualifications, and educational programs for teaching languages in European countries [47]. The reports indicate the important status of English in the European Framework of Reference for Languages. Russak [48] argues that researchers would not neglect the needs or rights of students with SEN who are required to learn English and other foreign languages according to policy document advocates (regarding inclusion and language education) as a large amount of research has shown that students with SEN might have more difficulties in linguistic functioning (in learning languages) than others [49,50,51]. For example, Andreou et al. ( [49] found that students with learning difficulties would be more likely to have academic problems, such as phonological awareness, and oral or written speech comprehension. Similar results were also found in Hong Kong, China. Ho and Fong [52] evidenced that the students with dyslexia performed significantly worse than those in the control group in nearly all English tests. Marashi and Dolatdoost [53] studied the relationships among Iranian EFL learners with ADHD and their speaking complexity, accuracy, and fluency (CAF). The research results revealed a significant negative correlation between ADHD and speaking complexity; this indicated that the higher the level of ADHD in learners, the less complicated their language. In this case, it is essential to pay more attention to those students with SEN in EFL learning. 

English is one of the most important foreign languages in the Chinese formal educational system, which might result in more challenges in the LRC tryouts. As part of the fast development of EFL education in China, English is emphasized from primary school to university; its important language status has influenced many Chinese students at all examination levels. As a foreign language, undoubtedly students with SEN experience more hardships during the learning process. The MOE [54] requires that students with SEN undertake English as one of the selective courses from year 7 in special schools. Meanwhile, English is required to be delivered to students with physical disabilities such as the deaf and blind in special schools and vocational schools. However, up until now, to our best knowledge, there has been no study investigating students with SEN learning English and teachers’ teaching practices in inclusive classrooms rather than in special schools on the Chinese mainland. By not paying much attention to those with SEN, it might be difficult for them to successfully transform to study at higher education levels [13]. Moreover, insufficient instructional practices for teachers will lead to low rates of acceptance for students with SEN to study in inclusive classrooms [48]. Thus, this paper examines the implementation of inclusion regulations and EFL teaching practices in the field. We used an exploratory survey adapted from Russak’s study [48], which focused on Iranian EFL teachers to see if their instructional practices and training reflected the requirements as delineated in inclusion regulations and EFL requirements. The following questions were asked:

1. Do EFL teaching practices in China reflect inclusion regulations and EFL policies?
2. What are the attitudes of EFL school teachers regarding the inclusion of students with SEN in inclusive classrooms?
3. Are there differences between teachers with and without training in special education in inclusion practices, support services provided, and attitudes toward inclusion of pupils with SEN in inclusive classrooms?

## 2. Method

### 2.1. Participants

There were 328 EFL school teachers who participated in the study. They were recruited from the three largest provinces in east, south, and central China between 2019 and 2020. After obtaining ethical approval (Zhejiang Normal University in April 202, no. 2SRT2020002) from the principal researcher’s university, the research assistant sent all the research information to the schools that were registered in the local EDB. With the permission of the EDB, the participants were recruited from the local Education Bureau (EDB) LRC programs or via snowball sampling. That is, one participant recommended another who met the required characteristics of the recruiting process [55]. The participants could obtain all the research information and consent forms from the local EDB website. Participants were informed that participation was voluntary, without personal identification, and that their data would be anonymous for the purpose of the research publication. The participants who signed the consent forms received an online survey. The background information of the participants is provided in Table 1. 

Table 1 shows that female teachers have major roles in the teaching profession in K12 schools in China. Most teachers are less than 40 years old with over five years of teaching experience. That is, they are not novice teachers. We should note that over 80% of participants reported that they never received training in special education in EFL teaching or other language teachings.

### 2.2. Instrument 

As the study was explorative at this stage, we designed the questionnaire based on Russak’s survey [48]; it was developed specifically for this study in the Chinese context. The questionnaire was originally designed in Chinese and then translated into English by using a back translation approach [56]. The questionnaire included four sections: background information about the teacher participants (six questions), inclusion practices in the EFL class (six questions), support services provided for pupils with SEN in the regular EFL class (five questions), and teachers’ attitudes concerning the inclusion of pupils with SEN in the regular EFL class (three questions). The details of the tool are shown in Appendix A. 

### 2.3. Statistical Analysis

First, descriptive statistics were conducted. In terms of categorical variables, we computed frequency counts and percentage scores; in terms of continuous variables, means and standard deviations were calculated. The chi-square test was then used to examine the associations between categorical variables. The binary choice logistic regression model was adopted to see each specific attitude. The confidence interval level was set at 95%. All analyses were performed using SPSS version 23. Finally, the open questions regarding benefits and weaknesses in inclusive classrooms, teachers’ attitudes toward students with SEN in LRC initiatives, and the reasons were analyzed by using a thematic analysis [57] via NVivo 12.

## 3. Results 

The present study examines if there are significant differences between teachers’ attitudes toward LRC of students with SEN in EFL teaching and learning across age, gender, school type, teaching experience, class type, and training in special education. Table 2 shows the findings regarding the inclusion of students in EFL classrooms. About two-thirds of teachers responded that students with SEN were taught in inclusive classrooms while one-third reported that students with SEN were not included. The mean number of pupils per class with each type of disability (LD, ADHD, dyslexia, autism, and other disabilities) was based on teacher responses to question items 10–14. The number of teacher responses to these questions was based primarily on teacher impressions rather than the specific numbers of disabilities from specialists. 

The support services provided for students with SEN in the regular EFL class are shown in Table 3.

The majority of teachers who taught students with SEN in the regular class reported that they did not have teaching assistants or companies, see Table 4. Further, the majority of teachers reported that students with SEN received the same number of English language instruction hours as the regular class and were taught with the same instructional materials. 

The chi-square test results showed that, regarding the attitudes toward the necessity of including SEN students in the LRC initiatives in EFL classrooms, the types of schools, classes, and special education training experiences for English teachers were associated with it. Teachers’ attitudes about whether SEN students should use the same EFL learning materials as normally-developed students were linked to the types of schools and their teaching experiences. Types of schools, teaching experiences, and training experiences in special education were associated with teachers’ attitudes toward the consistency of adopting different teaching methods between students with SEN and those who are normally developed. The types of schools were only associated with teachers’ attitudes regarding consistency in using the same amount of time in teaching students with SEN and those who are normally developed.

After the selection of factors associated with teachers’ attitudes toward inclusive education, we conducted the binary choice logistic regression model for each specific attitude. With each model, we only included the factors with the significant results according to the chi-square test (see Table 5). 

The logistic regression results suggest that when considering the effects of the type of school, class, and training experiences on the teachers’ attitudes toward the necessity of including SEN students in the LRC, only the school type and training experience were found to have significant impacts on the attitude. The logit model suggested the estimated coefficients were positive and significant, which meant that compared to the baseline, the probit of outcomes increased. In detail, middle school teachers showed more of a tendency to accept SEN students into the LRC. Likewise, teachers with previous special education training were more likely to have positive attitudes toward LRC initiatives. Regarding the attitudes about whether SEN students should use the same EFL learning materials as normally-developed students, middle school teachers demonstrated a more positive propensity compared to primary school teachers. Moreover, middle school teachers had more approving attitudes than primary school teachers toward the consistency of EFL teaching methods between SEN and normally-developed students, and so did the teachers with previous SEN training experiences compared to those without. In the comparison of primary school teachers, middle school teachers were more apt to believe that students with and without SEN might not need to learn EFL for the same amount of time per week.

The open questions were analyzed using a thematic analysis by Nvivo 12. We used sentiment analysis; we found that most of the teachers did not have positive attitudes toward the acceptance of students with SEN to learn EFL in inclusive classrooms. The details are shown in Table 6. 

We read the responses of the open questions carefully, categorized the units, compared the nodes, and grouped them into themes. Three main themes emerged:

Inclusive environment;
Teachers’ quality;
Satisfy students’ needs.

Most teachers did not have positive attitudes toward the acceptance of students with SEN in normal classrooms. An EFL learning environment was a major concern in the participants’ responses. One of the middle school teachers expressed that “English is a key subject for students in middle schools so that parents and principals had a high expectation on students’ scores in all kinds of examinations. If students with SEN were accepted into regular classrooms, they may feel difficult to catch up with other students. Consequently, they would have a low level of self-confidence and resilience”. Most teachers had concerns about the language learning progress of students with SEN, which was also seen as a weakness in this group of students. Both primary and middle school teachers believed that normally-developed students would learn EFL much faster than students with disabilities, particularly those with ADHD and autism. Moreover, teachers could not pay much attention to those with SEN as the normally-developed students might have been unsatisfied; consequently, it was difficult for teachers to control the classrooms. 

Other concerns involved the personal intellectual and/or physical development between students with SEN and normally developed students in learning EFL in an inclusive environment. A primary teacher concluded the following reasons:

“The inclusion policy for students to learn English in inclusive classrooms brings significant benefits for those who have been deprived rights before the implementation of LRC in China. The big leap helps students with SEN to have an equal opportunity to enjoy the nine-year compulsory education in mainland. However, we have to admit that students with SEN have their own characteristics. For example, a couple of students with ADHD would be very difficult to manage and often they make noises loud while others do exercise in the class. They might not be aware that their behavior would result in chaos, but teachers and other normally developed students felt very annoyed. Furthermore, my teaching performance, including students’ academic performance in examinations, strategies in classroom management, feedback from parents and students, would become a key KPI in my professional development. These assessments would influence on my promotion, and bonus. In this regard, I believe that many teachers, not only me, would not be happy to accept students with SEN in EFL classrooms in an inclusive environment.”

(Participant no. 30)

Teachers’ quality was another concern raised by teachers in their responses. Most EFL teachers reflected that they did not have training experience in special education. That is, they did not know the students’ intellectual and psychological traits, or the teaching strategies to manage them in an inclusive environment. A primary teacher said that she only joined in the pre-service teaching held by working schools, but it was a very simple and fast process. She only accessed very basic information concerning psychology counseling; she believed it might not have been enough. Other teachers mentioned that there was no specific information or program designed to help teachers in specific subjects to help students with SEN in inclusive classrooms. For example, a middle school teacher commented: “Specific education information or programs were only provided for graduates who studied in teachers’ colleges rather than other majors. Instead, most of EFL teachers in China are graduates from foreign language colleges/departments. This gap needs to be addressed by education managers and policy makers.” 

Some teachers also pointed out that it would be tough to satisfy their needs of students with SEN experiencing EFL in inclusive classrooms. A male teacher with more than 10 years of teaching experience expressed that he was not supportive of an inclusion policy at his workplace for the following reason:

“Students with SEN needs English teachers’ more attention and much care in a 45-min class. Moreover, students with SEN have their own needs for learning EFL. Some students who have a very light syndrome would require t”eachers to use the same teaching materials as they plan to join in college entrance examinations. However, others with serious issues, such as the deaf, might not have the same request. In this case, they are better to be rearranged in a small class with special teachers or assistants to help English teachers in a daily teaching session.”

(Participant no. 39)

Suggestions were also presented in terms of how to assist LRC initiatives in EFL classes, but they were part of a mixed trend. For example, using technology was popular among respondents. Teachers held positive attitudes toward the use of apps, E-tutors, and AI technologies to help students with SEN, besides the support of teachers. Teachers also recommended that small/neutral classes might be more beneficial for students with SEN as they have similar personal experiences and backgrounds, making it easy for them to communicate. However, some argued that technology cannot replace teachers in regular classrooms. They suggested that although students with SEN are not normally developed in their lives, peer communication in an inclusive environment is essential for socialization and, consequently, it benefits them in academic learning. 

## 4. Discussion

This study was an explorative study to examine (1) students with SEN in learning English as a foreign language and (2) teachers’ attitudes toward the inclusion of students with SEN in inclusive classrooms in China. The three research questions were fully answered. 

### 4.1. Do EFL Teaching Practices in China Reflect Inclusion Regulations and EFL Policies?

Based on the inclusive regulations and school policies, students with SEN should learn English in inclusive environments. School principals and teachers in K–12 have been required to implement the LRC initiative according to the requirement of MOE [13] in China. English as a foreign language was emphasized as important by the participants; whether the teaching practices were reflected in inclusive environments was not found in the study. This might be due to two reasons. First, the LRC initiative is a trial in many urban areas. That is, it has not been an inclusive law in the blueprints of all schools [13]. LRC needs to be implemented depending on the regional economy and educational resources. Due to the disparity of economic development, many rural and regional schools do not have enough facilities to accept students with SEN. The result is in line with Deng’s study [33], who indicated that there was a big gap between rural and urban schools in China, such as funding, resources, and qualified teachers. Second, although English is an important foreign language in K–12 schools, English is a selective subject in many public schools from year 3. However, English from K7 to K–12 is a compulsory subject in both public and private schools. In this case, teachers working at middle schools would face more challenges and pressures in their daily work lives if they implement the LRC initiative. 

Regarding the appropriate training for EFL teachers, it appears that students with SEN in mainland China are being taught, in many instances, by teachers with no training in special education or knowledge in the field of special education. Moreover, there are no promotions for the majority of teachers to take in sustained professional development programs. While this situation mirrors similar situations reported in other European countries where students with SEN are being taught additional languages in regular classes by teachers with training in special education [41,46,48,58], it shows that neither teachers nor school managers have paid much attention to the enhancement of the quality of education and the professional development of teachers, theoretically and practically. 

In terms of the support service, some students with SEN have been provided with enough support. However, very few resources are provided for students with SEN in inclusive classrooms while learning English in schools. The insufficient information is not beneficial for students with SEN in inclusive classrooms; the requirements of the MOE were not filled [13]. The current study indicates that the majority of students with SEN who used the same materials to learn EFL as the normally-developed students did not receive additional hours for studying EFL. The teachers reported that there were very few teaching assistants in the inclusive EFL class. However, participants also reported that accommodations could be adjusted for students with SEN who studied EFL in a regular class setting. These accommodations could be grouped into three categories: technology aids for EFL learning, personal attention and care, and modifications of the class materials. This result is similar to what Russak [48] evidenced in Israel—that these three aids would be considered in accommodations. 

Assistive technology is being increasingly used in special education to help disabled children in learning and in daily life [59,60]. Although technology could help the disabled to better their quality of life and communication in society [61], it requires application devices in the classroom, and assumes that teachers and pupils know how to use them appropriately. These concerns were also pointed out by Chinese scholars Yang and Huang [62], who claimed that low computer literacy and other barriers result in success in technological integration in the language learning classroom. These barriers include intrinsic and extrinsic difficulties, such as low levels of self-confidence, ICT proficiency, technical support, and system overload. 

Adjusting teaching materials has not been reflected in the study. This was not in agreement with the LRC initiative in Xiao’s study [37]. Modifying teaching materials to tailor to students’ needs is not only beneficial for those who are disabled to target their short-term and long-term learning goals, but it also enhances the motivation, self-efficacy, and self-esteem of students [63]. However, a teacher without special education training might think that by decreasing the workload, students with SEN could participate as best as they can. In practice, this might tell students that they are excluded and they are not good enough to finish activities similar to others in the classroom. This is very similar to showing personal care to students with SEN. In order to provide language skills and learning strategies for students with SEN, teachers should obtain specific knowledge in special education and acquire skills on how to teach students who are struggling and receive practical training and experience with teaching EFL to SEN populations. These were not seen positively in the study. 

### 4.2. Teachers’ Attitudes about Including Students with SEN in Learning English in Regular Classes

The findings of the study indicate that teachers have had mixed attitudes toward the inclusion of students with SEN in inclusive classrooms (regarding learning English). This ambiguity has been documented in previous studies and the results are equivocal [48,64,65,66]. Negative comments regarding the inclusion of students with SEN in the regular EFL class reflected teachers’ low levels of confidence and unsatisfied skills concerning special education as the main reasons. More specifically, teachers felt inadequate and insufficient to meet the needs of students with SEN within the framework of the regular EFL teaching. The teachers also felt that the students could not receive as much attention that they needed, resulting in slowing down the rest of the class. These results are in line with other research studies that evidenced that there was an urgent need to provide adequately support and resources for general education teachers [67,68].

The majority of teachers in this study expressed that students with SEN should be taught English either in a special class or in the LRC with appropriate help. The participants concluded that students with SEN need individual attention, care, and extra support from qualified teachers who have special education training, to learn EFL curriculum designed for students with SEN, and that they need to receive extra time inside and outside of regular class learning. Meanwhile, teachers felt that they could not really satisfy the needs of students with SEN in the regular class. Their reasons included the negative aspects mentioned above, such as the lack skills and knowledge regarding how to help.

Concerning the students with SEN in inclusive classrooms, there is a lack of time and resources. These findings echo the findings from Nijakowska (2014), which was based on teachers and teacher trainees in six European countries. Moreover, the results are similar to studies undertaken in China, where researchers argued about the quality of LRC due to the lack of resources and qualified special teachers and teaching assistants [11]. These findings are not unique in China. Research studies have also documented that EFL teachers faced similar challenges in managing students with SEN in Israel, the UK, and Greece [48,58,69]. 

### 4.3. The Relationships among Teacher Training, Practices, and Attitudes

Findings of the present study show that there are significant differences between primary and middle school teachers with and without special education concerning the teachers’ attitudes toward including students with SEN in regular classrooms, using the same teaching materials, adjusting teaching instructions, spending the same amount of time in learning EFL. Middle school teachers held more positive attitudes toward the four aspects than primary school teachers. This result is not in accordance with the study result in Israel [48]. It could be explained in two ways. First, the English subject for disabled students who are in inclusive classrooms is not compulsory in primary education (from year 1 to year 3). Moreover, there is no EFL framework or curriculum designed for special education. That is, most parents will desire to send their children to special schools if they are diagnosed with severe disabilities [52]. In this regard, there might not be so many cases in regular primary schools. Second, primary English teachers often only finish undergraduate study in English education or Teachers’ education, but middle school English teachers have to fulfill a lot of in-service teaching practices, and most of them are postgraduates in China. In this regard, they might obtain more knowledge of teaching strategies and coping skills to handle the challenges of students with SEN. As a result, this could increase their awareness in inclusive education, and enhance their self-confidence and self-efficacy. 

We should note the insignificant differences between teachers with and without training in teaching students with SEN—concerning their attitudes in using the same teaching materials and spending the same amount of time in teaching EFL to the rest of the class. Training in special education could help teachers obtain knowledge in special education, but training alone may not have an impact on teachers’ attitudes regarding the inclusion of pupils with SEN in inclusive classrooms [70]. Teachers’ professional support, resources, teaching experiences, and social networks might contribute to their attitudes toward the teaching strategies in EFL as well [6,48,69]. 

## 5. Limitations and Conclusions

We acknowledge two limitations of the study. First, the participants were all from central China rather than the different parts of the country. We used a number of teachers as the showcase; in the near future, this could assist us in developing the research into a large-scale study in China. Second, the English subject is very unique for Chinese students, particularly those who are disabled. Rather than courses taught in the mother tongue, the English language could bring more challenges to students with SEN compared to mathematics, science, and chemistry. We hope that more foreign language teachers, such as those who teach German, Spanish, and Japanese, will be involved, i.e., to see if they have different attitudes and behavioral patterns in comparison to English teachers in China. 

This study is the first exploratory study to investigate K–12 EFL teachers’ attitudes toward including students with SEN in inclusive classrooms. Findings of the current study are in line with conclusions driven by other countries where certain aspects of the inclusion regulations and language learning requirements are being implemented, while others are not. Teachers have missed attitudes in various aspects, but they believe that the crucial determinants of the success of inclusive language learning are support services and teacher training. There is an urgent need for the development of EFL teaching materials and a learning framework for students with SEN in inclusive classrooms. Moreover, principals, teachers, and decision makers have to pay more attention to the Chinese LRC plan in different types of schools and regions. Professional development programs and workshops are also needed for both experienced and new teachers. 

## Figures and Tables

**Table 1:** Background information of the participants.

Items	N
1. Gender	
Female	215
Male	113
2. Age	
20–29	99
30–39	168
40–49	52
50–59	9
3. School types	
Primary School	254
Middle School	74
4. Years of teaching EFL:	
1–5	97
6–10	146
Over 10	85
5. Which type of classes are you teaching?	
Regular Class	265
Small Groups	52
Inclusive Class	11
6. Did you complete any training in special education for EFL teachers?	
Yes	62
No	266

**Table 2:** Inclusion of pupils with SEN in regular EFL classes.

	Mean	SD
Inclusion of students with SEN in regular class N (%)		
Yes 215 (66) No 113 (34)		
Number of students with dysgnosia per class	1.1	1.1
Number of pupils with ADHD per class	1.3	1.6
Number of pupils with Autism per class	0.9	0.9
Number of pupils with LD per class	1.4	1.7
Number of pupils with other disabilities per class	1.3	0.9

**Table 3:** Support services provided for students with SEN in the regular EFL class.

Questions Items	Yes (N)	No (N)
15. Are the students with SEN provided with the same teaching materials to learn EFL as those in the regular class?	211	117
16. Are the students with SEN provided with the same teaching methods to learn EFL as those in the regular class?	202	126
17. Do the students with SEN learn EFL for the same number of hours per week as those in the regular class?	200	128
20. Do you have any teaching assistants or company teachers in your class?	180	148

**Table 4:** The results of the chi-square test.

	English Teachers’ Attitudes toward Inclusive Education			
	9. Are students with special needs included in the regular EFL class?	15. Do the students with special educational needs learn EFL according to the same materials?	16. Do the students with special educational needs learn EFL according to the same instructional practice?	17. Do the students with special educational needs learn EFL for the same number of hours per week as the students in the regular class?
Factors				
1. Gender	0.513	1.906	0.383	3.543
2. Age	1.493	0.623	1.621	2.498
3. Type of school	8.510 **	6.714 **	13.298 **	14.124 **
4. Teaching experience	1.963	7.699 *	8.297 *	3.528
5. Class	11.404 **	3.197	6.685	6.552
6. Training experience	6.155 *	1.468	3.907 *	0.003

Note: ** *p* < 0.01, * *p* < 0.05.

**Table 5:** Binary choice model to investigate the effects of teachers’ individual factors on their attitudes toward inclusive education.

	English Teachers’ Attitudes toward Inclusive Education			
	9. Are students with special needs included in the regular EFL class?	15. Do the students with special educational needs learn EFL according to the same materials?	16. Do the students with special educational needs learn EFL according to the same instructional practice?	17. Do the students with special educational needs learn EFL for the same number of hours per week as the students in the regular class?
Factors				
3. Type of school (baseline: primary school)				
Middle school	0.896 **	0.654 **	1.107 **	1.148 **
4. Teaching experience (baseline: one-to-five years)				
Six-to-ten years		−0.522	−0.407	
Over ten years		0.139	0.380	
5. Class (baseline: normal class)				
Small-scale class	0.600			
Special class	1.626			
6. Training experience (baseline: no training)				
training	0.781 *		0.685 *	
R^2^	0.09	0.06	0.10	0.06

Note: estimates in the table presented the lg of each outcome’s probit of 1 when compared to the 0 probit. ** *p* < 0.01, * *p* < 0.05.

**Table 6:** Sentiment analysis.

Codes	Number of Coding References
Total	166
Mixed	45
Negative	21
Neutral	58
Positive	42

## Data Availability

The data presented in this study are available on request from the corresponding author. The data are not publicly available due to ethical requirement.

## Appendix A

**Survey for EFL Teachers**

Background information

1. Gender
  - Female
  - Male
2. Age
  - 20–29
  - 30–39
  - 40–49
  - 50–59
3. School types
  - Primary School
  - Middle School
4. Years of teaching EFL:
  - 1–5
  - 6–10
  - Over 10
5. Which type of classes are you teaching?
  - Regular Class
  - Small Groups
  - Inclusive Special Needs Class
6. Did you complete any training in special education for EFL teachers?
  - Yes
  - No
7. If Yes, please describe the training you received____________________________
8. Please describe the inclusive policy in your school:
  ______________________________________________________________
9. Do you agree that students with SEN should be accepted into regular EFL classrooms?
10. How many students with dysgnosia are in your class? [Diagnosed on the basis of a neurological, psycho-didactic, or didactic evaluation]? [Response is numerical]
11. How many students with ADHD are in your class? [Diagnosed on the basis of a neurological, psycho-didactic, or didactic evaluation]? [Response is numerical]
12. How many students with Autism are in your class? [Diagnosed on the basis of a neurological, psycho-didactic, or didactic evaluation]? [Response is numerical]
13. How many students with LD are in your class? [Diagnosed on the basis of a neurological, psycho-didactic, or didactic evaluation]? [Response is numerical]
14. Do you have students with other types of disabilities [like physical disabilities, PDD, Asperger’s, etc.] in your EFL class?
15. Do the students with special educational needs learn EFL according to the same materials? Yes/No.
16. Do the students with special educational needs learn EFL according to the same instructional practice? Yes/No.
17. Do the students with special educational needs learn EFL for the same number of hours per week as the students in the regular class? Yes/No.

**Open questions**

18. If pupils with special educational needs are allowed accommodations to testing conditions, such as extra time, having their tests read to them, or doing a different test from the regular class, please list the accommodations recognized and implemented in your school.
19. If pupils with special educational needs are allowed accommodations or modifications to the learning environment in class, such as using a computer, getting handouts of the material written on the board, only doing a portion of the assigned classwork please describe them.
20. Do you have a teaching assistant in your class? Yes/No If so, explain why.
21. What are the positive aspects of having pupils with special educational needs in the regular EFL class?
22. What are the negative aspects of having pupils with special educational needs in the regular EFL class?
23. In your opinion, should pupils with special educational needs be taught EFL in the regular class or in special EFL classes? Yes/No Explain your answer.
